# Screening Methods for Downy Mildew Resistance in Maize: A Systematic Review

**DOI:** 10.3390/genes17030350

**Published:** 2026-03-22

**Authors:** Mable Chebichii Kipkoech, Arsenio Ndeve, Joao Bila, Pedro Fato, Suwilanji Nanyangwe, Kolawole Peter Oladiran, Constantino Francisco Lhamine

**Affiliations:** 1 Department of Crop Production, Faculty of Agronomy and Forest Engineering, Eduardo Mondlane University (UEM), Maputo P.O. Box 257, Mozambique; mablekipkoech13@gmail.com (M.C.K.); ndevegod@gmail.com (A.N.); suwilanjinanyangwe02@gmail.com (S.N.); oladirankolawole5@gmail.com (K.P.O.); 2 Centre of Excellence in Agri-Food Systems and Nutrition (CE-AFSN), Eduardo Mondlane University, 5° Andar, Edificio da Reitoria, Praça 25 de Junho, Maputo P.O. Box 257, Mozambique; 3 Department of Crop Protection, Faculty of Agronomy and Forest Engineering, Eduardo Mondlane University (UEM), Maputo P.O. Box 257, Mozambique; jbilay@gmail.com; 4 Instituto de Investigação Agrária de Moçambique (IIAM), Maputo P.O. Box 3558, Mozambique; bycosta.francisco@gmail.com

**Keywords:** downy mildew, maize resistance, screening methods, *P. sorghi*, spreader row, quantitative trait loci, marker-assisted selection, disease incidence, systematic review, phenotypic screening

## Abstract

**Background/Objectives:** Downy mildew, caused by *Peronosclerospora* and *Sclerophthora* species, is a major constraint to maize production in tropical and subtropical regions, with yield losses of 30–100%. This systematic review synthesised evidence on methods used to screen maize for downy mildew resistance and assessed their effectiveness, reliability, and associated markers. **Methods:** PubMed, Google Scholar, ScienceDirect, and CAB Abstracts were searched (last searched 22 October 2025) for English-language studies (1990–2025) evaluating phenotypic or molecular screening methods. Risk of bias was assessed using the RoB 2 framework. Narrative synthesis was conducted following a protocol registered on the Open Science Framework. **Results:** Twelve studies met the inclusion criteria, predominantly from India and Cambodia. Spreader row systems (seven studies) and conidial spray inoculation (six studies) were the most common field methods, while the glasshouse sandwich technique generated the highest disease pressure. Cross-method correlations were strong (r = 0.92–0.99), and heritability estimates ranged from 0.50 to 0.97. QTL mapping identified resistance loci on chromosomes 2, 3, and 6, with chromosome 6 stable across multiple pathogen species. Evidence certainty was moderate for method effectiveness and low for molecular markers. **Conclusions:** Established phenotypic screening methods reliably discriminate resistant germplasm; however, standardised protocols, broader geographic validation, and independent molecular marker confirmation are needed.

## 1. Introduction

Maize (*Zea mays* L.) occupies a central position in global agriculture as a principal source of food, livestock feed, and industrial raw materials, with annual production surpassing one billion metric tons across a wide range of agro-ecological zones [1,2]. Ranked as the third most cultivated cereal after rice and wheat, the crop underpins food security in developing regions while sustaining livestock value chains and diverse industrial applications—from biofuel production to processed consumer goods—in more advanced economies [3,4]. Nevertheless, realising the full productive potential of maize remains a persistent challenge, as biotic stresses, notably pests and diseases, continue to constrain yields across major production areas worldwide [2,5].

Among the array of biotic threats, downy mildew (DM) stands out as one of the most destructive fungal diseases of maize, particularly in tropical and subtropical environments where warm, humid conditions favour pathogen development [6,7]. The disease is caused by obligate oomycete pathogens of the genera Peronosclerospora and Sclerophthora, encompassing several species of economic significance: *Peronosclerospora sorghi* (sorghum downy mildew), *P. philippinensis* (Philippine downy mildew), *P. maydis* (Java downy mildew), *P. sacchari* (sugarcane downy mildew), *P. heteropogoni* (Rajasthan downy mildew), and *Sclerophthora rayssiae* var. *zeae* (brown stripe downy mildew) [6,7,8,9]. These pathogens display pronounced host specificity and geographic partitioning; for instance, *P. sorghi* infects both maize and sorghum in peninsular India, whereas *P. heteropogoni* persists through collateral hosts such as *Heteropogon contortus* in Rajasthan [7,9,10].

Geographically, DM pathogens are distributed across all major maize-growing continents. Asia, which accounts for approximately 28% of global maize output, harbours widespread DM occurrence in countries, including China, India, Indonesia, Nepal, Pakistan, the Philippines, Thailand, and Vietnam, where epiphytotics frequently develop under conditions of high humidity, cool temperatures (21–23 °C), and intermittent drizzle [1,5,9]. The disease has also been documented in several African countries—Mozambique, Nigeria, Uganda, and Zaire—as well as in parts of Latin America, North America, Europe, Australia, and Egypt, confirming its status as a global phytosanitary concern [9,11].

The economic toll of DM is substantial. Yield losses in affected areas range from 30% to total crop failure, with the severest impacts recorded in resource-limited tropical lowlands, subtropics, mid-altitude, and highland environments [6,9,12,13,14]. Systemic infection at the seedling stage triggers a constellation of debilitating symptoms—stunted growth, leaf chlorosis and mottling, white-striped foliage, excessive tillering, and deformed reproductive organs—that frequently culminate in premature plant death or barren ears [1,2]. Pathogen dissemination proceeds through multiple channels, including soil-borne oospores, airborne conidia, and contaminated seed, all of which amplify disease spread under intensive cropping systems [1]. In Indonesia, for example, DM caused by *P. maydis*, *P. philippinensis*, and *P. sorghi* has materially reduced maize productivity in key production zones, such as North Sumatra, where output reached 1.83 million tons in 2020 despite persistent biotic pressure [5,15].

A range of management strategies has been deployed against DM, encompassing cultural practices (deep ploughing, rogueing infected plants, and adjusting sowing dates), chemical seed treatments with systemic fungicides such as metalaxyl, and integrated pest management frameworks [2,7,16]. However, sustained dependence on fungicides raises significant concerns regarding cost, environmental contamination, and the emergence of pathogen resistance, rendering chemical control an unsustainable long-term option, particularly for resource-poor farming communities [9,17]. In this context, host plant resistance has emerged as the most cost-effective and ecologically sound alternative, as demonstrated by successful resistance breeding programmes in Asia and sub-Saharan Africa [7,11,18]. Genetic studies indicate that DM resistance in maize is polygenic, governed by additive gene effects and quantitative trait loci (QTLs) mapped to chromosomes 1, 2, 3, 6, 7, 9, and 10, which can be dissected using recombinant inbred lines (RILs) and molecular marker platforms, including restriction fragment length polymorphisms (RFLPs) and simple sequence repeats (SSRs) [1,6,8].

The success of resistance breeding, however, hinges fundamentally on the availability of reliable screening methods for identifying resistant germplasm. Phenotypic screening approaches include both field-based techniques—such as infector row planting, conidial spray inoculation, and spreader row systems—and controlled-environment protocols like direct seed inoculation of pre-germinated seeds under glasshouse conditions [3,11,19,20,21]. These methods differ considerably in their efficiency and applicability; infector row planting ensures consistent pathogen exposure under field conditions, while seedling-stage spray inoculation can achieve up to 100% disease incidence in controlled environments [3,22]. Complementing phenotypic evaluation, molecular screening through QTL mapping, positional cloning, and transcriptome profiling enables the identification of resistance-associated markers and candidate genes, thereby facilitating marker-assisted selection (MAS) for durable, broad-spectrum resistance across diverse pathogen strains and environments [1,6,8]. Nonetheless, methodological heterogeneity, environmental variability, and pathogen diversity continue to impede the standardisation and cross-study comparability of screening outcomes [7,9].

Despite noteworthy advances in DM resistance breeding—including the release of resistant open-pollinated varieties (OPVs), elite inbred lines (e.g., the CIMMYT CML series), and commercial hybrids—a critical gap persists in the systematic synthesis and comparative evaluation of screening protocols and their performance across diverse pathogen–environment combinations [7,9,18]. Existing studies have employed a wide array of phenotypic and molecular approaches; yet, no comprehensive review has collated and appraised the relative strengths, limitations, and contextual suitability of these methods. This fragmentation limits the ability of breeders and pathologists to select optimal screening strategies and hampers efforts to establish standardised protocols for resistance evaluation.

This systematic review addresses these gaps by synthesising the available evidence on methods used for screening downy mildew resistance in maize and assessing their effectiveness, reliability, and applicability. Specifically, the review is guided by the following research questions: (1) What are the most commonly used methods to screen maize for downy mildew resistance? (2) How effective and reliable are these screening techniques under different environmental and pathogen conditions? (3) What phenotypic or molecular markers are associated with resistance?

## 2. Methods

### 2.1. Review Protocol and Reporting Standards

This systematic review was conducted in accordance with the Preferred Reporting Items for Systematic Reviews and Meta-Analyses (PRISMA) guidelines and followed a protocol registered prospectively in the Open Science Framework (Registration DOI: 10.17605/OSF.IO/U8B25). Adherence to this pre-registered protocol ensured transparency in study selection, data extraction, and synthesis throughout the review process.

### 2.2. Eligibility Criteria

Studies were eligible for inclusion if they evaluated maize (*Zea mays* L.) germplasm, inbred lines, hybrids, or breeding populations for resistance to downy mildew pathogens, including *P. sorghi*, *P. philippinensis*, *P. maydis*, *P. heteropogoni*, or related species [11]. Eligible studies employed phenotypic screening approaches—such as field-based infector rows or glasshouse spray inoculation—or molecular methods, including QTL mapping and marker-assisted selection [10,23]. Comparators encompassed evaluations conducted across contrasting environments (e.g., tropical lowlands versus subtropical zones) or against distinct pathogen strains, as well as repeated-trial assessments of method reliability. Primary outcomes of interest included infection incidence rates, false-positive and false-negative minimisation, reproducibility across trials, and scalability for large-scale breeding. Secondary outcomes encompassed standardised protocols and region-specific adaptations that enhance transferability. Only peer-reviewed primary research (experimental and observational) and reviews or meta-analyses published in English between January 1990 and October 2025 were considered.

Studies focusing exclusively on non-maize hosts without direct comparisons to *Zea mays* were excluded, as were investigations concerned solely with pathogen epidemiology, fungicide efficacy, or cultural management practices that did not evaluate resistance screening. Grey literature, conference abstracts, theses, and unpublished reports lacking full methodological detail were also excluded to uphold analytical rigour [24]. Duplicate publications, retracted articles, and studies with insufficient outcome data or methodological descriptions were omitted to prevent redundancy and minimise bias in the evidence synthesis.

For the purposes of synthesis, included studies were grouped thematically according to screening approach—phenotypic field-based methods, controlled-environment (glasshouse) protocols, and molecular or genomic techniques—and further stratified by pathogen species and geographic context to facilitate comparative evaluation of method performance.

### 2.3. Information Sources

A systematic literature search was conducted to identify all relevant studies on screening methods for downy mildew resistance in maize, following the Preferred Reporting Items for Systematic Reviews and Meta-Analyses extension for searching (PRISMA-S) to ensure transparency and reproducibility in evidence retrieval [24,25]. The search strategy was designed to capture the full spectrum of phenotypic and molecular screening approaches reported across diverse agroecological settings, encompassing early field-based inoculation techniques developed for tropical environments [11] through to contemporary genomic tools employed for QTL identification [1,6].

Four electronic databases were searched, each selected for its complementary disciplinary coverage: PubMed, for molecular biology and plant pathology literature; Google Scholar, for broad multidisciplinary indexing spanning peer-reviewed and grey literature; ScienceDirect, for applied science and engineering perspectives; and CAB Abstracts, for specialised agricultural and crop protection research [26,27]. All four databases were last searched on 22 October 2025. In addition, the reference lists of all included studies and relevant review articles were hand-searched to identify any further eligible records not captured by the electronic searches.

### 2.4. Search Strategy

The search employed a sensitive Boolean syntax combining Medical Subject Headings (MeSH) in PubMed with free-text terms to accommodate terminological variation across the literature. The core query was structured as follows: (“maize” OR “Zea mays”) AND (“downy mildew” OR “Peronosclerospora” OR “Sclerophthora”) AND (“screening” OR “resistance” OR “inoculation” OR “QTL” OR “marker”) AND (“method*” OR “technique*” OR “protocol*”). Truncation symbols (e.g., “method*”) were applied to capture synonyms and plurals, while proximity operators were adapted to the search syntax of each database. Searches were restricted to English-language publications, reflecting the predominance of maize breeding research published in English and practical constraints on translation resources [24]. The temporal scope ranged from 1990 to 2025, consistent with the eligibility criteria, thereby encompassing seminal epidemiological overviews alongside more recent investigations of pathogen strain variability and molecular marker development. No additional filters for study design or publication type were applied at the search stage so as to maximise recall.

### 2.5. Selection Process

Study selection proceeded through two sequential stages. In the first stage, two reviewers independently screened all titles and abstracts using Rayyan, a web-based platform that supports blinded assessments, keyword highlighting, and automated duplicate detection, thereby promoting efficiency without compromising transparency [28]. Each record was classified as potentially eligible, clearly ineligible, or uncertain; records marked as uncertain by either reviewer were retained for full-text evaluation. In the second stage, the same two reviewers independently assessed the full texts of all remaining records. Disagreements arising at either stage were resolved through structured consensus discussion; where agreement could not be reached, a third reviewer served as an impartial arbitrator, an approach consistent with methodological guidance on minimising selection error and subjectivity in systematic reviews [24,29]. No automation tools beyond Rayyan were used at any point in the selection process.

**Figure 1:**
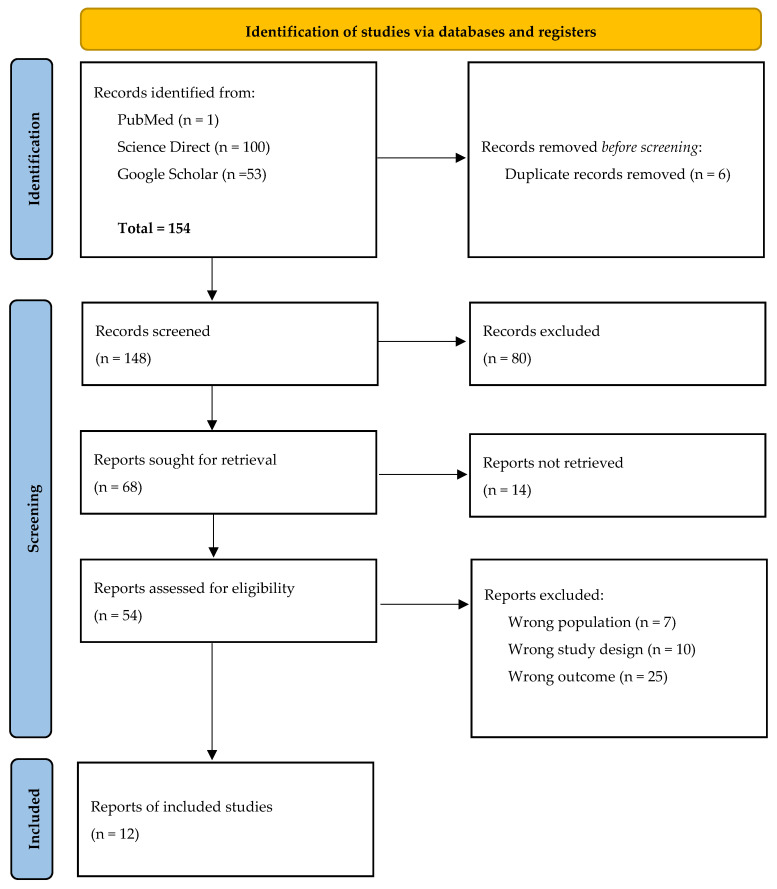
PRISMA flow diagram showing the study selection process.

The entire selection workflow is documented in a PRISMA flow diagram (Figure 1), which reports the total number of records identified from each database, the number screened at the title and abstract level, those assessed at full text, and the final number of studies included in the synthesis, together with explicit reasons for exclusion at each stage [24].

### 2.6. Data Collection Process

Data from the 12 included studies were extracted using a structured form developed in Microsoft Excel, following recommendations for maintaining consistency and traceability in systematic reviews [30]. Prior to full-scale extraction, the form was refined through a calibration exercise in which both reviewers independently extracted data from three studies, compared their results, and discussed discrepancies to clarify coding definitions and resolve ambiguities [31]. Two reviewers then independently extracted data from each eligible report, working in separate copies of the spreadsheet without access to each other’s entries so as to minimise confirmation bias. Upon completion, the two datasets were cross-checked item by item; disagreements—for example, differences in how a screening method was categorised or conflicting interpretations of reported disease incidence thresholds—were resolved through discussion, with a third reviewer consulted where consensus could not be reached [24].

Where key data elements were missing, unclear, or ambiguously reported—such as incomplete inoculation protocol descriptions, unreported environmental conditions during screening trials, or unclear marker nomenclature—supplementary materials and appendices were reviewed, and related publications from the same research groups were cross-referenced to fill information gaps. For studies in which critical methodological details remained unavailable after these efforts, the missing information was recorded in the extraction form and flagged during synthesis so that it could be addressed as a limitation in the interpretation of findings [30]. No automation tools were employed during data extraction; all data collection was performed manually to permit careful contextual interpretation of each study’s methods and results, given the considerable heterogeneity in screening approaches, pathogen species, and geographical contexts across the included literature.

### 2.7. Data Items

#### 2.7.1. Outcomes

Data were sought for all results compatible with two outcome domains. The primary outcome domain encompassed method effectiveness, operationalised as disease incidence rates, false-positive and false-negative rates, and any quantitative metrics of screening accuracy reported by the original authors. The secondary outcome domain comprised method reliability and applicability, defined as inter-trial reproducibility (e.g., correlation coefficients across repeated experiments), protocol standardisation, and scalability for large-scale breeding programmes. Where a study reported multiple measures within the same outcome domain—for instance, disease incidence at different time points or under different inoculation pressures—all compatible results were extracted to preserve the breadth of available evidence [1,6].

#### 2.7.2. Other Variables

Beyond outcome data, the following study-level and methodological variables were extracted: author(s) and publication year; geographical location of the study; maize germplasm type (inbred lines, hybrids, open-pollinated varieties, or mapping populations); downy mildew pathogen species; screening method category (phenotypic field-based, controlled-environment, or molecular/genomic); specific inoculation technique (e.g., conidial spray, direct seed inoculation, or infector row planting); environmental conditions under which screening was conducted (humidity, temperature, and field versus glasshouse setting); identified phenotypic or molecular markers, including QTL positions on specific chromosomes; and funding sources where reported [30]. For variables that were missing or ambiguously reported, the assumptions and handling procedures described in Section 2.6 were applied, and each instance was documented in the extraction form to ensure transparency during synthesis.

### 2.8. Study Risk of Bias Assessment

The risk of bias assessment for the included studies was performed systematically to evaluate the methodological quality and potential sources of bias, ensuring the robustness of the evidence synthesised in this review on screening methods for downy mildew resistance in maize. This process followed established guidelines for assessing bias in non-randomised studies of interventions and observational research in plant pathology and agricultural sciences [32]. A customised risk of bias tool was developed, adapted from the ROBINS-I framework (Risk Of Bias In Non-randomised Studies—of Interventions), which is suitable for evaluating studies involving field trials, greenhouse experiments, and genetic analyses [32]. The tool assessed domains such as selection bias (e.g., representativeness of maize genotypes and pathogen strains), performance bias (e.g., consistency in screening methods like spreader rows or artificial inoculation), detection bias (e.g., objectivity in scoring disease incidence or severity), attrition bias (e.g., completeness of data across environmental or pathogen conditions), and reporting bias (e.g., selective outcome reporting for phenotypic or molecular markers). Each domain was rated as low, some concerns, or high, with justifications documented for transparency.

Two independent reviewers evaluated the risk of bias for each of the 12 included studies. Reviewers worked independently using a standardised extraction form in Microsoft Excel, which facilitated blinded assessments and automated flagging of discrepancies. Following individual assessments, results were compared, and any disagreements were resolved through consensus discussions. In instances of unresolved differences, a third reviewer provided arbitration to ensure impartiality, aligning with recommendations for minimising subjectivity in quality appraisals [33]. No automation tools were employed beyond basic spreadsheet functions, as the assessment required specific judgement on context-specific elements like environmental variability in field screenings and pathogen strain specificity. The overall risk of bias ratings was integrated into the evidence synthesis to contextualise findings on screening methods’ effectiveness, reliability, and associated markers, with sensitivity analyses conducted where high-risk studies might influence conclusions.

### 2.9. Effect Measures

Given the nature of the included studies, which were drawn from plant pathology and resistance breeding rather than clinical intervention research, conventional epidemiological effect measures such as risk ratios or mean differences were not applicable. Instead, effect measures were defined according to the outcome domains and analytical approaches characteristic of downy mildew (DM) screening in maize. The predominant effect measure across the 12 included studies was percentage disease incidence, typically recorded between 21 and 34 days after inoculation or sowing and classified against categorical resistance scales. Although the precise thresholds varied among studies, most adopted broadly comparable frameworks in which resistant genotypes exhibited less than 10% incidence and highly susceptible entries exceeded 50–76% [2,3,7,9]. Where analysis of variance was reported, genotype effects were tested using F-statistics, with significance typically declared at *p* < 0.01, and mean separation was performed using least significant difference (LSD) or Duncan’s multiple range test (DMRT) [7,8,11].

For studies employing quantitative genetic analyses, broad-sense heritability (h^2^) served as a key effect measure for gauging the proportion of phenotypic variance attributable to genetic differences among genotypes, with reported estimates ranging from 0.50 to 0.97 across different pathogen species and screening environments [6,7,9,33]. Pearson correlation coefficients (r) were used to assess the concordance between screening methods or environments; notably, Arulselvi and Selvi [3] reported r = 0.99 between field and glasshouse incidence, while Jadhav et al. [33] obtained r = 0.949 (*p* < 0.01) between field and controlled-environment evaluations, and Rashid et al. [7] found r = 0.922 (*p* < 0.01) between sorghum and Rajasthan downy mildew field trials. These correlation-based measures provided a basis for evaluating the transferability and consistency of screening outcomes across methodological settings.

In studies that integrated molecular approaches, quantitative trait locus (QTL) mapping employed logarithm of the odds (LOD) scores and the proportion of phenotypic variance explained (PVE) as the principal effect measures for identifying genomic regions associated with DM resistance. LOD thresholds of 2.5 to 3.0 at α = 0.05 were adopted as significance cut-offs [1,6,33]. Among the mapped loci, Kim et al. [1] reported seven QTLs, with qDM4 attaining the highest LOD of 18.16 and qDM1 explaining 12.95% of phenotypic variance, while George et al. [6] identified a stable QTL on chromosome 6 with R^2^ values of 13–31% and a likelihood ratio of 56.7 (*p* < 0.01) across five pathogen species. Jadhav et al. [33] detected a chromosome 3 QTL with LOD scores ranging from 5.3 to 18.1 and PVE of 14–19%, validated across three environments. Complementing these mapping efforts, gene expression analyses quantified fold-change differences between resistant and susceptible genotypes; Kim et al. [8], for instance, documented expression levels of 1050–2338% in resistant lines relative to 93–133% in susceptible entries, with significance confirmed by quantitative real-time PCR (*p* < 0.01).

Beyond statistical effect measures, several performance metrics were extracted to evaluate the practical reliability of screening protocols. Coefficients of variation (CV) ranged from 14% to 98% across studies, reflecting varying degrees of environmental noise and methodological standardisation [6,7,9,11]. Reproducibility across seasons or years was quantified by Yen et al. [9], who reported 88% reproducibility for sorghum downy mildew and 76% for Rajasthan downy mildew screening outcomes. Susceptible check performance served as an internal validity indicator, with entries such as CM500 consistently achieving 92–100% incidence across multiple studies [2,3,7,8,9]. Table 1a,b shows the summary of the Effect Measures for downy mildew screening in maize.

### 2.10. Synthesis Methods

#### 2.10.1. Eligibility for Each Synthesis

Studies were allocated to specific syntheses on the basis of the screening approach employed and the outcome domain reported. Following data extraction, the intervention characteristics of each study, namely screening method category (phenotypic field-based, controlled-environment, or molecular/genomic), pathogen species evaluated, and germplasm type tested, were tabulated and compared against the planned groupings established in the eligibility criteria (See Table 1). Studies that employed more than one screening approach were assigned to each relevant synthesis group. Where a study reported outcomes for multiple pathogen species, its results were disaggregated by species and entered into the corresponding subgroup. This tabulation step allowed the reviewers to verify that each synthesis contained a coherent set of studies addressing a shared methodological question and to identify any gaps in coverage prior to commencing the narrative synthesis [24,30].

#### 2.10.2. Data Preparation

Several preparatory steps were undertaken before synthesis. Disease incidence values reported on differing categorical scales were harmonised to a common percentage-based metric to enable cross-study comparison; where authors employed ordinal severity scores (e.g., 0–9 scales), the corresponding percentage incidence thresholds published in the same study were used to map entries into the standardised resistance categories of highly resistant (0%), resistant (≤10%), moderately resistant (11–25%), susceptible (26–50%), and highly susceptible (>50%), consistent with the classifications adopted in the majority of included studies [7,9]. For studies that reported QTL mapping results, LOD scores, phenotypic variance explained (PVE), and marker positions were extracted as reported, with no further transformation applied. Where summary statistics such as coefficients of variation, heritability estimates, or correlation coefficients were absent from the main text but recoverable from Supplementary Table S1 or Figure S1, these values were calculated or transcribed accordingly. Missing data that could not be resolved through the procedures outlined in Section 2.6 were recorded as “not reported” and clearly flagged in the summary tables to ensure transparency during interpretation [30].

#### 2.10.3. Tabulation and Visual Display

Results from individual studies were presented using a structured summary table (See Table 1). Each table recorded the key study characteristics, effect measures (percentage incidence, heritability, correlation coefficients, LOD scores, and PVE), and performance metrics (CV, check line incidence, and reproducibility indices) to permit direct visual comparison across studies. A PRISMA flow diagram (See Figure 1) was used to depict the study selection process [24].

#### 2.10.4. Synthesis Approach

A narrative synthesis was adopted as the primary method of integrating findings, and no formal meta-analysis was conducted. This decision was driven by the pronounced clinical and methodological heterogeneity among the included studies, which differed substantially in pathogen species, inoculation techniques, environmental conditions, germplasm backgrounds, resistance rating scales, and outcome reporting conventions [6,7,9]. Under such conditions, pooling effect estimates into a single summary statistic would risk producing a misleading composite that obscures meaningful variation across screening contexts. The narrative synthesis was structured thematically around three pillars: phenotypic field-based screening methods, controlled-environment (glasshouse) protocols, and molecular or genomic approaches. Within each thematic group, studies were compared with respect to method effectiveness (disease incidence ranges, false-positive and false-negative minimisation), reliability (heritability, inter-trial reproducibility, and cross-method correlation), and applicability (scalability, cost implications, and adaptability to different agroecological settings). Convergent and divergent findings were highlighted to draw out overarching patterns and identify areas where evidence was insufficient or contradictory [30].

#### 2.10.5. Exploration of Heterogeneity

Although formal meta-regression was not performed, possible sources of heterogeneity among study results were explored through structured subgroup comparisons. Studies were stratified by pathogen species (e.g., sorghum downy mildew versus Rajasthan downy mildew versus Java downy mildew), screening environment (field versus glasshouse), geographic region (South Asia, Southeast Asia, and sub-Saharan Africa), and germplasm type (inbred lines, hybrids, and open-pollinated varieties). Within each stratum, the direction and magnitude of effect measures were compared to assess whether differences in these variables could account for observed variation in screening outcomes. For instance, the differential reproducibility reported for sorghum downy mildew (88%) and Rajasthan downy mildew (76%) screening by Yen et al. [9] was examined alongside the cross-species correlation of r = 0.922 reported by Rashid et al. [7] to determine whether pathogen identity influenced the consistency of field-based evaluations. Similarly, the high field–glasshouse correlation (r = 0.99) documented by Arulselvi and Selvi [3] was contrasted with less tightly correlated results from other studies to investigate whether the screening environment modulated method agreement.

#### 2.10.6. Sensitivity Analyses

In the absence of a formal meta-analysis, the sensitivity of the synthesised findings was assessed qualitatively. The robustness of the principal conclusions was examined by considering whether they would change if studies with the highest risk of bias, the smallest sample sizes, or the most atypical environmental conditions were excluded from the narrative synthesis. Particular attention was paid to the influence of studies reporting unusually high coefficients of variation (e.g., CV up to 98% in George et al. 2003 [6]), which could introduce disproportionate noise into the overall assessment of screening reliability. Additionally, the consistency of resistance classifications was checked by comparing outcomes for common reference genotypes and susceptible checks (e.g., CM500) across independent studies; the near-universal attainment of 92–100% incidence in CM500 across multiple laboratories and field sites [2,3,7,8,9] served as an internal validation benchmark against which the stability of the broader findings was judged.

### 2.11. Reporting Bias Assessment

The risk of bias arising from missing results in the synthesis was assessed at two levels. At the study level, the completeness of outcome reporting was evaluated during data extraction: each study was checked for selective reporting by comparing the outcomes described in the methods section against those presented in the results, and any discrepancies were documented in the extraction form [30]. At the synthesis level, the potential for publication bias was considered qualitatively, given that the small and heterogeneous pool of 12 included studies precluded the use of funnel plots or statistical tests such as Egger’s regression, which require a minimum of approximately ten homogeneous effect estimates to yield meaningful results [24]. To mitigate the influence of reporting bias, the search strategy deliberately encompassed a broad range of databases, including CAB Abstracts for specialised agricultural literature, and imposed no restrictions on study design at the search stage so as to maximise recall of all available evidence. The possibility that studies reporting null or unfavourable screening outcomes may be underrepresented in the published literature was acknowledged as a limitation and is addressed in the discussion.

### 2.12. Certainty Assessment

The certainty of evidence for each principal outcome was appraised using the Grading of Recommendations, Assessment, Development and Evaluations (GRADE) framework, adapted for the non-clinical context of agricultural screening research [30]. Each body of evidence was initially rated as high certainty and subsequently downgraded by one or two levels based on five domains: risk of bias within individual studies, inconsistency of results across studies, indirectness of evidence relative to the review question, imprecision of effect estimates, and suspected publication bias. Upgrading was considered where large and consistent effect sizes, evidence of a dose–response gradient, or plausible confounders that would have diminished an observed effect were identified. For each outcome domain—method effectiveness, method reliability, and marker–resistance association—a summary certainty rating of high, moderate, low, or very low was assigned to communicate the degree of confidence that the true effect lies close to the estimate reported in the synthesis [24].

## 3. Results

### 3.1. Study Selection

The systematic database search across PubMed, Google Scholar, ScienceDirect, and CAB Abstracts yielded a combined total of 154 records. After automated and manual removal of six duplicate citations, 148 unique records remained for title and abstract screening. At this initial stage, two independent reviewers working in Rayyan classified each record as potentially eligible, clearly ineligible, or uncertain. Following blinded evaluation, 80 records were excluded on the basis of irrelevant subject matter, non-maize crop focus, or absence of any screening or resistance evaluation component, leaving 68 reports for full-text assessment.

During full-text evaluation, each of the 68 reports was appraised against the predefined eligibility criteria (Section 2.2) by the same two reviewers working independently. Of these, 56 reports were excluded for the reasons detailed below and summarised in Table 2, yielding a final set of 12 studies that met all inclusion criteria and were retained for qualitative synthesis. No additional eligible studies were identified through hand-searching of reference lists. The complete selection workflow, from initial identification through to final inclusion, is depicted in the PRISMA flow diagram (Figure 1) [24].

The excluded reports were classified into three mutually exclusive categories based on the primary reason for exclusion (Table 2). Seven reports were excluded for the wrong population: these studies focused on non-maize hosts or on mixed-crop systems in which maize-specific resistance screening was not the central objective. For example, Anahosur and Hegde [19] and Narayana et al. [22] investigated downy mildew inoculation protocols on sorghum rather than maize, while Carlsson et al. [21] and Odvody and Frederiksen [16] addressed pathogen biology on alternative hosts. Mushayi et al. [13] and Susilowati et al. [15] similarly examined downy mildew in non-maize or tangentially related cropping contexts, and Madhu et al. [37] centred on a model organism outside the scope of this review.

Ten reports were excluded for wrong study design, as they comprised reviews, methodological guidelines, or conceptual papers that did not present primary field-based or laboratory-based phenotypic or molecular screening data. This category included widely cited systematic review methodology references such as Page et al. [24], Higgins et al. [30], and Rethlefsen et al. [25], as well as software and tool descriptions by Ouzzani et al. [28] and Waffenschmidt et al. [29]. Although these works informed the methodological framework of the present review, they did not themselves report empirical resistance screening outcomes and were therefore ineligible for inclusion in the evidence synthesis.

The largest group of exclusions, 25 reports, fell under the wrong outcome. These studies addressed aspects of downy mildew biology, genetic mapping, QTL analysis, disease epidemiology, or molecular genetics that, while related to the broader field, did not directly evaluate or compare screening methods for resistance identification in maize. Notable examples include Agrama et al. [23] and George et al. [6], which focused on QTL mapping and genetic architecture of resistance without systematic assessment of screening methodology, and Yen et al. [9] and Kim et al. [1,8], which reported resistance evaluations but framed primarily around pathogen characterisation or gene expression rather than methodological comparison. Rashid et al. [18] and Cardwell et al. [38] were similarly excluded because their principal contribution lay in epidemiological surveillance or germplasm characterisation rather than in the appraisal of screening protocols per se.

### 3.2. Study Characteristics

**Figure 2:**
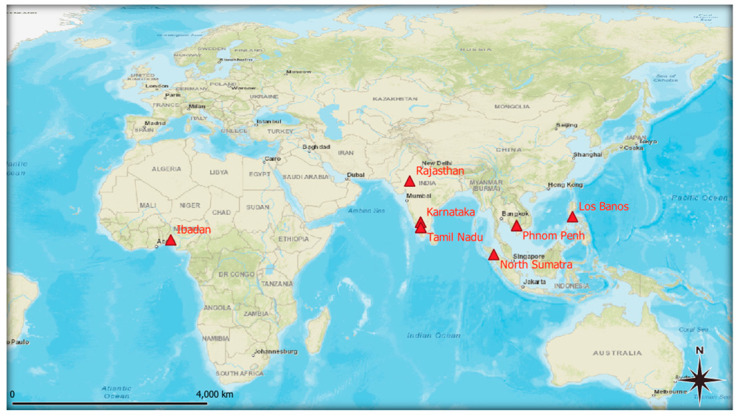
Geographical distribution of studies on screening methods for downy mildew resistance in maize.

The 12 included studies are summarised in Table 3 and Figure 2. Geographically, India contributed the largest share (five studies conducted at hotspot locations, including Mandya, Coimbatore, and Udaipur) [3,6,7,9,35], followed by Cambodia [1,8], with single studies from Nigeria [11], Indonesia [36], and the Philippines [34]. Jadhav et al. [33] and Sumathi et al. [2] conducted trials at multiple Indian sites. This distribution reflects the concentration of downy mildew pressure in South and Southeast Asia, while the Nigerian study broadened the environmental diversity of the evidence base.

Field-based phenotypic techniques predominated. Spreader row systems were the most widely adopted method, reported in seven studies [2,6,8,9,11,33,36], while artificial conidial spray inoculation was employed by six [2,3,11,33,34,35]. Controlled-environment protocols included the glasshouse sandwich technique [3,7] and whorl inoculation under field conditions [6,7,9]. Molecular approaches complemented phenotyping in five studies: QTL mapping via SSR and RFLP markers [1,6,7,33], qRT-PCR expression profiling [1,8], and combined SSR/SNP marker analysis [36]. The remaining studies relied exclusively on phenotypic evaluation [2,11,34,35].

Most field trials were established under conditions favouring DM development—relative humidity exceeding 85%, night temperatures of 20–23 °C, and intermittent rainfall [7,11]. George et al. [6] tested germplasm across five Asian sites with differing climatic profiles, while Djaenuddin et al. [36] confirmed resistance stability across Indonesian greenhouse and open-field environments. Glasshouse protocols afforded tighter environmental control, with Rashid et al. [7] reporting the sandwich technique as yielding the highest and most consistent disease pressure. Regarding pathogen diversity, the most frequently evaluated species were *P. sorghi* and *P. heteropogoni*. Rashid et al. [7] and Yen et al. [9] screened against both sorghum and Rajasthan downy mildew, finding that SDM-resistant lines were frequently, though not invariably, resistant to RDM. George et al. [6] extended coverage to five species, identifying a chromosome 6 QTL with broad-spectrum stability, and Kim et al. [1] mapped QTLs for *P. sorghi*, *P. maydis*, and *Sclerophthora macrospora*.

Percentage disease incidence, assessed 21–34 days after inoculation, was the standard phenotypic outcome across all 12 studies, with genotypes typically classified as resistant (<10% incidence) or susceptible (>50%) [7,9]. Additional phenotypic indicators included suppression of systemic infection and chlorosis [11,35], reduced lesion size and sporulation [34], and SPAD chlorophyll readings [33]. Key resistant reference genotypes included UMI 935(w) [3], Ki3 and CML228 [6,8], and CML433 [7]. At the molecular level, QTLs were mapped to chromosomes 2, 3, and 6 [6,33,36], with flanking markers bnlg420, umc1165, bnlg1297, umc2353, and phi098 validated across environments [1,33]. Candidate resistance genes bZIP33, Bak1, and Ppr were nominated through differential expression analysis [8]. Rashid et al. [7] and Yen et al. [9] reported overlapping QTL positions for SDM and RDM, suggesting shared genetic control of resistance to multiple pathogen species.

### 3.3. Risk of Bias in Studies

**Figure 3:**
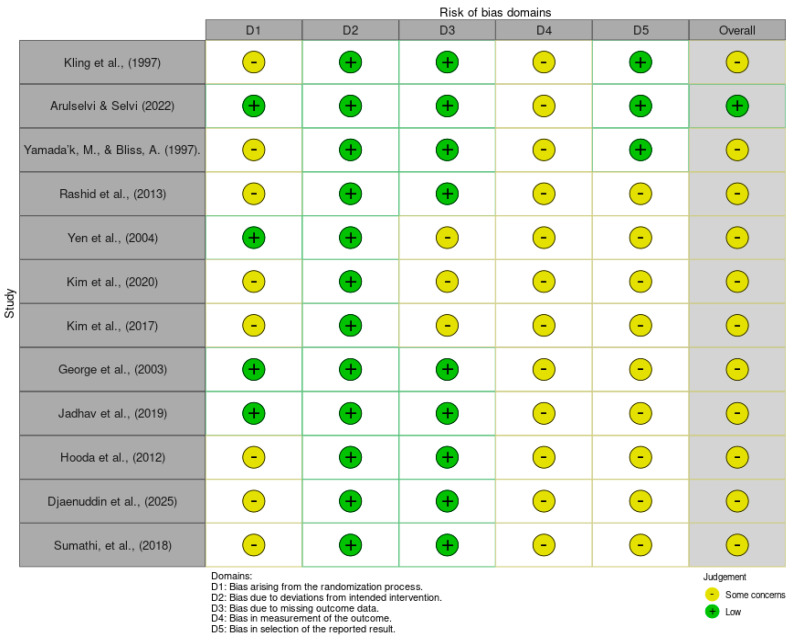
Risk of bias assessment using the Robvis online tool.

Risk of bias was assessed for each of the 12 included studies using the Cochrane Risk of Bias 2 (RoB 2) framework, adapted for the agricultural screening context of this review [32]. Judgements were made across five domains: bias arising from the randomisation process (D1), bias due to deviations from intended interventions (D2), bias due to missing outcome data (D3), bias in measurement of the outcome (D4), and bias in selection of the reported result (D5). The domain-level and overall judgements for each study are presented in Table 4 and Figure 3. No study was rated as having a high overall risk of bias; one study was judged to have low overall risk, while the remaining eleven were rated as raising some concerns.

Regarding randomisation (D1), only four studies, Arulselvi and Selvi [3], Yen et al. [9], George et al. [6], and Jadhav et al. [33], were rated as low risk, having described randomised or systematic plot allocation procedures. The remaining eight studies [1,2,7,8,11,34,35,36] raised some concerns because details of randomisation were either incompletely reported or absent, making it difficult to rule out allocation-related confounding.

All 12 studies were judged to have a low risk of bias due to deviations from intended interventions (D2). The screening protocols described in each study—whether field-based spreader rows, glasshouse sandwich inoculation, or molecular assays—were applied consistently to all test genotypes within a given experiment, with no evidence of protocol deviations or selective treatment application that might have differentially affected outcome measurement.

For missing outcome data (D3), the majority of studies were rated as low risk, having reported complete or near-complete datasets for all evaluated genotypes. Three studies raised some concerns in this domain: Yen et al. [9] did not fully account for genotype attrition across seasons, while Kim et al. [8] and Kim et al. [1] presented partial expression or incidence data for certain genotypes without explaining the reasons for missingness, leaving open the possibility of outcome-dependent data loss.

Bias in measurement of the outcome (D4) was the domain most frequently flagged. All 12 studies were rated as raising some concerns, primarily because disease incidence was assessed visually by field or laboratory personnel whose blinding status was not reported. Visual scoring of downy mildew symptoms—percentage incidence, severity scales, or lesion size—is inherently subjective, and none of the included studies described measures to mask assessors to genotype identity during evaluation. This limitation is endemic to phenotypic plant pathology research but warrants acknowledgement as a potential source of differential misclassification, particularly in field trials where border effects and spatial variation in inoculum pressure may further confound visual assessments [6,7].

Bias in selection of the reported result (D5) was rated as low for only two studies—Kling et al. [11] and Arulselvi and Selvi [3], which presented outcomes consistent with their stated objectives and appeared to report all pre-specified analyses. The remaining ten studies raised some concerns because pre-registered analysis plans were not available, and it was not possible to determine whether the reported outcomes and analytical choices (e.g., selection of time points, resistance thresholds, or QTL significance cut-offs) were specified prior to data inspection or selected post hoc from among multiple possible analyses [1,7,9].

### 3.4. Results of Individual Studies

#### 3.4.1. Phenotypic Field-Based Screening

Kling et al. [11] evaluated maize genotypes in Nigeria using field spray inoculation, seedling incubator methods, and spreader rows. Disease incidence among test entries ranged from 0.1% to 41.4%, whereas susceptible checks exhibited 6–94% incidence (LSD = 13.1, *p* < 0.001). The incubator method achieved less than 5% disease escape, indicating strong inoculation pressure. Coefficients of variation ranged from 31% to 35%, reflecting moderate field-level noise. In India, Arulselvi and Selvi [3] compared field sick-plot and glasshouse screening, reporting near-perfect concordance between the two environments (r = 0.99). The resistant line UMI 935(w) recorded 0–2.6% incidence, while the susceptible check CM500 reached 92–100%. The glasshouse protocol was judged the most efficient overall for discriminating resistant from susceptible entries.

Yamada and Aday [34] optimised artificial inoculation parameters in the Philippines, identifying a combination of 0.5-leaf-stage seedlings and 50 × 10^3^ conidia/mL as optimal for maximising the differential between resistant and susceptible genotypes (50–58% difference). Highly significant effects were detected for leaf stage (F = 77.27), conidial density (F = 20.31), and plant material (F = 71.67), all at *p* < 0.01. Hooda et al. [35] screened germplasm at three Indian hotspots against both *P. sorghi* and *P. heteropogoni*, identifying three inbred lines with consistent resistance (8.6–13.9% incidence) across two years and both pathogen species, while CM500 attained 100% incidence in all trials. Sumathi et al. [2] employed spreader rows and early-morning conidial inoculation (3:30–4:30 AM) in a sick-plot environment during Rabi 2013, with 16 of 22 progenies recording 0% incidence and CM500 again reaching 100%, confirming adequate inoculum pressure.

Djaenuddin et al. [36] conducted multi-region field trials in Indonesia under natural inoculum pressure supplemented by artificial inoculation, scoring severity on a 0–9 scale. Downy mildew incidence ranged from 20% to 86% across accessions, with the best-performing entry (05022-00847) recording 20% and four accessions falling below 28%. Spreader rows achieved greater than 80% infection in susceptible material within two weeks of symptom onset, confirming the efficacy of the field protocol.

#### 3.4.2. Combined Field and Controlled-Environment Screening

Rashid et al. [7] compared the glasshouse sandwich technique with field-based infector row and whorl inoculation methods in India, evaluating germplasm against both sorghum downy mildew (SDM) and Rajasthan downy mildew (RDM). The sandwich method generated the highest and most uniform disease pressure, with susceptible checks attaining 93.6–97.2% incidence and 100% infection in spill-over rows. Cross-method correlation between SDM and RDM field trials was strong (r = 0.922, *p* < 0.01). Broad-sense heritability ranged from 0.65 to 0.97, and 14 inbred lines recorded less than 18.8% incidence, with CML-433 achieving 0%. Genotype effects were highly significant (*p* < 0.01), with a genotype × method interaction detected at *p* < 0.05. CVs ranged from 14% to 20%.

Yen et al. [9] employed the sandwich technique for SDM and whorl inoculation for RDM across tropical and subtropical Indian sites (Mandya and Udaipur). Five lines proved resistant to both pathogens (0–8.6% incidence), with CM500 reaching 99–100%. Heritability estimates were 0.75 for SDM and 0.63 for RDM. Genotype and year × genotype interaction effects were both highly significant (*p* = 0.0001). Reproducibility across seasons was 88% for SDM and 76% for RDM, with CVs of 19–30%. Jadhav et al. [33] combined spreader rows, seedling spray, and artificial epiphytotic protocols across field and glasshouse environments at Coimbatore and Mandya, reporting a field–glasshouse correlation of r = 0.949 (*p* < 0.01). Heritability was high (87–97%), and a chromosome 3 QTL (LOD = 5.3–18.1, PVE = 14–19%) flanked by marker bnlg420 was validated across all three environments

#### 3.4.3. Molecular and Genomic Screening

George et al. [6] tested germplasm across five Asian sites using spreader rows, artificial inoculation, and modified spreader row protocols, complemented by QTL mapping with SSR and RFLP markers. Six QTLs were identified, with the chromosome 6 locus exhibiting the strongest association (likelihood ratio = 56.7, *p* < 0.01; R^2^ = 13–31%) and stability across all five pathogen species evaluated. The resistant check Ki3 recorded 0–32% incidence, while CML138 reached 50–100%. Heritability ranged from 0.50 to 0.75, and CVs varied widely (15–98%), reflecting heterogeneity across sites and seasons.

Kim et al. [8] used spreader rows, QTL analysis, and RT-PCR in Cambodia, identifying high resistance in CML228, Ki3, and Ki11 under favourable disease conditions. Gene expression in resistant genotypes was markedly elevated (1050–2338% of baseline) compared with susceptible lines (93–133%), with significance confirmed at *p* < 0.01 and a cross-season correlation of r = 0.98. Susceptible checks reached 100% incidence in both seasons, and five candidate resistance genes were identified, including bZIP33, Bak1, and Ppr. Kim et al. [1] extended this work through qRT-PCR and QTL analysis, mapping seven QTLs for DM resistance in a recombinant inbred population. The strongest associations were detected at qDM4 (LOD = 18.16) and qDM1 (LOD = 14.12, PVE = 12.95%), with all seven QTLs validated and 15 genes upregulated in resistant genotypes (*p* < 0.001). Susceptible parents B73 and CML270 recorded 100% incidence, and the mapped QTLs collectively accounted for 65.8% of the genome under investigation.

The physical map positions of all reported QTLs, aligned to the B73 RefGen_v5 assembly, are presented in Table 5.

The chromosome 6 QTLs reported by George et al. (2003; bin 6.01, ~4.5–25.0 Mb) [6] and Djaenuddin et al. [36]; bin 6.01–6.02, ~4.5–90.0 Mb) show substantial positional overlap within the proximal region of chromosome 6. The George et al. interval is entirely contained within the broader Djaenuddin et al. interval, strongly suggesting that they detect the same underlying resistance locus. Additionally, qDM6 from Kim et al. [1] maps to chromosome 6 and may overlap with this region, although the resolution of flanking markers in the original study is insufficient to confirm co-localisation. By contrast, the chromosome 3 QTL identified by Jadhav et al. [33]; bin 3.05–3.06, ~155.3–165.8 Mb) represents a genetically independent resistance locus. The Rashid et al. [7] and Yen et al. [9] studies reported phenotypic co-segregation of SDM and RDM resistance consistent with shared genetic control, but did not provide marker-based physical positions, precluding formal overlap analysis. Fine-mapping studies using high-density SNP arrays or whole-genome sequencing in diverse genetic backgrounds are needed to definitively resolve whether the chromosome 6 signals represent a single pleiotropic locus or multiple tightly linked genes.

### 3.5. Results of Syntheses

#### 3.5.1. Overview of Contributing Studies

The narrative synthesis was structured around three thematic groups: phenotypic field-based screening (nine contributing studies), controlled-environment protocols (four studies, three of which also contributed to the field-based group), and molecular or genomic approaches (five studies). Across all groups, the overall risk of bias was moderate: eleven studies were rated as raising some concerns, and one was rated as low risk (Section 3.3). The most pervasive sources of concern were the absence of assessor blinding during visual disease scoring (D4, all 12 studies) and the lack of pre-registered analysis plans (D5, 10 studies). No study was rated as high risk in any individual domain.

#### 3.5.2. Synthesis of Screening Method Effectiveness and Reliability

No formal meta-analysis was performed owing to the pronounced methodological and contextual heterogeneity among included studies. The narrative synthesis revealed several convergent patterns. First, across all phenotypic methods, susceptible check genotypes (particularly CM500) consistently achieved 92–100% incidence, confirming that inoculation pressure was adequate in the vast majority of trials [2,3,7,8,9]. Second, field-based spreader rows and controlled-environment sandwich inoculation emerged as the two most effective protocols for discriminating resistant from susceptible germplasm: spreader rows were the most widely validated field method, while the sandwich technique generated the highest and most uniform disease pressure under glasshouse conditions [7]. Third, cross-method and cross-environment correlations were consistently high. Arulselvi and Selvi [3] reported r = 0.99 between field and glasshouse incidence, Jadhav et al. [33] obtained r = 0.949 between field and controlled environments, and Rashid et al. [7] found r = 0.922 between SDM and RDM field trials, indicating strong transferability of resistance rankings across settings.

Broad-sense heritability estimates for DM incidence were moderate to high across studies, ranging from 0.50 [6] to 0.97 [7,33], suggesting that a substantial proportion of phenotypic variation was genetically determined and thus amenable to selection. Seasonal reproducibility, quantified by Yen et al. [9], was 88% for SDM screening and 76% for RDM, indicating that while field phenotyping was broadly repeatable, pathogen-specific variation introduced a degree of inter-season inconsistency that breeders should account for in multi-year evaluation designs.

At the molecular level, QTL mapping consistently identified resistance-associated loci on chromosomes 2, 3, and 6, with the chromosome 6 region emerging as the most stable across pathogen species and environments. George et al. [6] detected a chromosome 6 QTL explaining 13–31% of phenotypic variance across five pathogen species, Jadhav et al. [33] validated a chromosome 3 QTL (PVE = 14–19%) across three environments, and Kim et al. [1] identified seven QTLs with LOD scores of 3.0–18.16. Candidate gene analyses by Kim et al. [8] nominated bZIP33, Bak1, and Ppr as functionally relevant, with expression fold-changes of 1050–2338% in resistant genotypes. These molecular findings complement phenotypic screening by offering marker-based tools for early-generation selection independent of environmental conditions.

#### 3.5.3. Sources of Heterogeneity

Subgroup comparisons revealed three principal sources of heterogeneity. First, pathogen species influenced screening consistency: SDM screening was more reproducible (88%) than RDM (76%) across seasons [9], and lines resistant to SDM were frequently but not invariably resistant to RDM [7,9], indicating that pathogen identity modulates the stability of resistance rankings. Second, screening environment contributed to variation in precision: CVs were consistently lower in glasshouse-based studies (14–20%) than in multi-site field trials (15–98%), with the extreme upper values driven largely by site-to-site heterogeneity in George et al. [6]. Third, geographic region and associated climatic conditions influenced disease pressure; the Indian and Cambodian sites that maintained optimal conditions for DM development (>85% RH, 20–23 °C) produced the most discriminating phenotypic separations, whereas Indonesian trials under variable field conditions yielded a broader incidence range (20–86%) with less sharp differentiation between resistance classes [36].

#### 3.5.4. Sensitivity Analysis

The robustness of the principal findings was assessed by considering the effect of excluding studies with the highest risk-of-bias concerns or the most atypical environmental conditions. Removing George et al. [6], which reported CVs of up to 98%, did not alter the conclusion that chromosome 6 harbours a major resistance QTL, as the same region was independently identified by Djaenuddin et al. [36] and corroborated by marker–trait associations in Jadhav et al. [33]. Excluding the two oldest studies [11,34] did not change the overall pattern of high susceptible-check performance or the superiority of the sandwich and spreader row methods, both of which were confirmed by more recent work [2,7]. The consistency of CM500 as a susceptible reference (92–100% incidence across five independent studies) further supported the stability of the synthesised conclusions, as this benchmark was unaffected by variation in pathogen species, geographic location, or screening protocol.

### 3.6. Reporting Biases

Assessment of reporting bias was conducted at both the study and synthesis levels. At the study level, comparison of stated objectives and reported outcomes revealed no clear evidence of selective outcome reporting: all 12 studies presented results for their primary stated endpoints, and no study reported a change in outcome definition between methods and results sections [30]. However, the absence of publicly available pre-registered protocols for all but the present review means that selective reporting cannot be definitively excluded, particularly with respect to the choice of resistance thresholds, time points for incidence assessment, and QTL significance cut-offs.

At the synthesis level, the small and heterogeneous pool of 12 studies precluded the use of funnel plots and any formal statistical tests for publication bias [24]. Several observations, nonetheless, raise the possibility that the published literature may over-represent positive findings. All included studies identified at least some resistant genotypes, and none reported a screening method that entirely failed to discriminate between resistance classes. Moreover, three of the five molecular studies reported QTLs with moderate to large effect sizes (PVE = 12–31%), and no study reported null QTL mapping results. While these patterns may reflect the genuine efficacy of the screening methods employed, they are also consistent with a publication landscape in which null or inconclusive findings are less likely to reach peer-reviewed journals. The broad database coverage adopted in the search strategy—including CAB Abstracts for specialised agricultural literature—was designed to mitigate this risk.

### 3.7. Certainty of Evidence

The certainty of evidence was appraised for three outcome domains using the GRADE framework adapted for agricultural research, as described in Section 2.12.

For method effectiveness (discrimination between resistant and susceptible genotypes), the evidence was rated as moderate. The body of evidence was drawn from 12 studies with broadly consistent findings—susceptible checks reliably achieved 92–100% incidence, and resistant genotypes consistently fell below 10%—supporting a clear direction of effect. However, the certainty was downgraded by one level for risk of bias, owing to the universal absence of assessor blinding during visual disease scoring (D4) and the lack of pre-registered analytical protocols in the majority of studies (D5). No further downgrade was applied for inconsistency, as the direction of the effect was uniform despite variation in magnitude, nor for indirectness, as the studies directly addressed the review question.

For method reliability (reproducibility and cross-method concordance), the evidence was rated as moderate. High cross-environment correlations (r = 0.92–0.99) and moderate-to-high heritability estimates (h^2^ = 0.50–0.97) across multiple studies provided strong support for the reliability of both field-based and glasshouse screening. The certainty was downgraded by one level for imprecision, reflecting the wide range of CVs reported (14–98%) and the differential reproducibility between SDM (88%) and RDM (76%) screening documented by Yen et al. [9]. Publication bias could not be formally assessed and therefore did not contribute to a further downgrade, though its potential influence was noted.

For marker–resistance association (identification of QTLs and candidate genes linked to DM resistance), the evidence was rated as low. Five studies contributed molecular data—George et al. [6], Jadhav et al. [33], Kim et al. [8], Kim et al. [1], and Djaenuddin et al. [36], collectively identifying QTLs on chromosomes 2, 3, and 6 and nominating several candidate genes. The low certainty rating for marker–resistance association reflects primarily limitations in study design rather than evidence of marker instability per se. Specifically, the certainty was downgraded by one level for risk of bias, owing to the absence of pre-registered analysis plans in all five contributing studies, which raises the possibility that QTL significance thresholds, marker selection criteria, or reporting of effect sizes may have been optimised post hoc. A further downgrade for imprecision was applied because QTL effect sizes varied substantially across studies and environments (PVE = 12–31% for the chromosome 6 locus), and the total number of independent mapping populations was small (*n* = 5). Importantly, this imprecision may reflect limited statistical power in individual studies rather than genuine biological instability of the markers: the chromosome 6 QTL was independently detected across five pathogen species [6] and confirmed by a geographically independent study [36], suggesting that the underlying genetic signal is robust. We therefore conclude that the low certainty rating is attributable to methodological limitations in the existing evidence base—particularly small sample sizes, absence of analytical pre-registration, and lack of independent validation cohorts—rather than to demonstrated failure of the markers to replicate across populations.

## 4. Discussion

### 4.1. General Interpretation of Results

This systematic review synthesised evidence from 12 studies spanning nearly five decades of research on screening methods for downy mildew (DM) resistance in maize. Three principal findings emerged from the synthesis. First, field-based spreader row systems and controlled-environment sandwich inoculation constitute the most widely validated and effective protocols for phenotypic resistance screening, consistently achieving adequate disease pressure—as evidenced by susceptible check incidence of 92–100% across independent laboratories and field sites [2,3,7,8,9]. Second, the high cross-method and cross-environment correlations documented in this review (r = 0.92–0.99) indicate that resistance rankings are broadly transferable between field and glasshouse settings, an observation of considerable practical significance for breeding programmes operating under varying resource constraints [3,7,33]. Third, molecular screening through QTL mapping has identified genomic regions on chromosomes 2, 3, and 6 that are consistently associated with DM resistance, with the chromosome 6 locus displaying stability across multiple pathogen species and environments [6,33,36].

These findings align with the broader plant pathology literature, which has long recognised that effective disease resistance screening requires both reliable inoculation systems and heritable host responses. The predominance of spreader row methodology in the included studies mirrors its established use in other cereal pathosystems, where the technique is valued for generating spatially uniform inoculum pressure under field conditions [20]. Similarly, the high heritability estimates reported across studies (h^2^ = 0.50–0.97) are consistent with earlier characterisations of DM resistance as a quantitatively inherited trait governed by additive gene effects [1,6], supporting the premise that phenotypic selection can yield meaningful genetic gain per cycle. The identification of chromosome 6 as a major resistance-associated region corroborates independent mapping efforts and suggests that this locus may harbour broadly effective defence genes amenable to marker-assisted introgression [6].

To assess whether the chromosome 6 QTLs reported across studies represent the same genomic interval, we aligned the flanking marker positions to the B73 RefGen_v5 assembly using coordinates cross-referenced from MaizeGDB (Table 5). The George et al. [6] chromosome 6 QTL (bin 6.01, approximately 4.5–25.0 Mb) and the Djaenuddin et al. [36] chromosome 6 locus (bin 6.01–6.02, approximately 4.5–90.0 Mb) show substantial positional overlap, with the George et al. interval entirely contained within the broader Djaenuddin et al. interval. This strongly suggests that both studies detected the same underlying resistance locus. Additionally, qDM6 from Kim et al. [1] maps to chromosome 6 and may overlap with this region, although the resolution of flanking markers is insufficient to confirm co-localisation. By contrast, the chromosome 3 QTL identified by Jadhav et al. [33] (bin 3.05–3.06, approximately 155.3–165.8 Mb, flanked by bnlg420 and phi073) represents a genetically independent resistance locus validated across three environments. The Rashid et al. [7] and Yen et al. [9] studies reported phenotypic co-segregation of SDM and RDM resistance, consistent with shared genetic control, but provided no marker-based physical positions, precluding formal overlap analysis.

The practical concordance between field and glasshouse screening outcomes is particularly noteworthy. In many tropical breeding programmes, field screening is constrained by seasonal availability of inoculum, unpredictable weather patterns, and logistical costs associated with maintaining sick plots across multiple locations [7,9]. The near-perfect field–glasshouse correlation reported by Arulselvi and Selvi [3] and the strong correspondence observed by Jadhav et al. [33] suggest that glasshouse-based protocols can serve as reliable surrogates during off-season periods or at sites where natural inoculum pressure is insufficient, thereby accelerating the screening cycle without compromising the accuracy of resistance classification.

However, the high cross-method correlations documented in this review (r = 0.92–0.99) warrant scrutiny regarding alternative explanations. First, the use of common susceptible checks (e.g., CM500) that anchor both endpoints of the incidence distribution may mechanically inflate correlation coefficients by ensuring that both extreme-susceptible and extreme-resistant entries are consistently ranked, regardless of assessment method. Second, co-location of field and glasshouse trials at the same research stations means that shared environmental conditions—temperature, humidity, and inoculum source—may contribute to concordance beyond what would be observed across geographically distant sites. Third, the possibility that the same unblinded assessors scored disease in both environments cannot be excluded, introducing potential expectation bias. Nevertheless, the consistency of resistance rankings across geographically distant sites (India, Cambodia, and Nigeria) and against distinct pathogen species supports genuine biological transferability as the principal explanation for the observed correlations.

From a practical standpoint, the two most effective screening methods differ markedly in resource requirements and throughput. Field-based spreader row screening is characterised by low infrastructure costs, high throughput (hundreds to thousands of entries per season in a single sick-plot), and seasonal dependency. By contrast, the glasshouse sandwich technique entails higher fixed infrastructure costs but offers year-round screening capability independent of seasonal constraints. On a per-sample basis, spreader row screening is substantially less expensive where field infrastructure already exists, whereas the sandwich technique offers superior cost-efficiency when amortised across multiple screening cycles per year or when field inoculum pressure is unreliable. None of the included studies reported formal cost-per-sample data; comparative cost–benefit analysis of DM screening methods is therefore identified as a priority for future operational research.

### 4.2. Limitations of the Evidence

Several limitations of the included evidence warrant careful consideration. The most pervasive concern is the absence of assessor blinding across all 12 studies. Visual scoring of DM symptoms—percentage incidence, severity ratings, and lesion dimensions—is inherently subjective, and unblinded assessors may be influenced, consciously or otherwise, by expectations regarding genotype performance. This limitation is endemic to phenotypic plant pathology research; yet, its potential to introduce differential misclassification should not be underestimated, particularly in field trials where spatial variation in inoculum pressure and border effects add further noise to visual assessments [6,7].

Critically, the near-perfect field–glasshouse correlation (r = 0.99) reported by Arulselvi and Selvi [3] should be interpreted with caution, given that the same unblinded assessors scored disease incidence in both environments. Expectation bias—whereby assessors familiar with genotype identity may unconsciously assign consistent ratings across settings—could inflate the apparent concordance beyond its true value. Similarly, the r = 0.922 between SDM and RDM field trials [7] may be subject to analogous bias if the same personnel scored both pathosystems. Without an independent blinded assessment, the magnitude of these correlations, though encouraging, cannot be taken at face value as evidence of purely biological transferability of resistance rankings. Future phenotypic screening studies should therefore adopt blinded outcome assessment protocols, digital image-based phenotyping using unmanned aerial vehicle (UAV) imagery coupled with machine learning-based disease quantification, or double-blind scoring by independent assessors masked to genotype identity, to strengthen the reliability of cross-method comparisons.

A second limitation concerns the narrow geographic representation of the evidence base. Five of the twelve studies were conducted in India, two in Cambodia, and the remaining five across Nigeria, Indonesia, and the Philippines. Sub-Saharan Africa—where DM is documented in Mozambique, Nigeria, Uganda, and Zaire [9,11]—was represented by only a single Nigerian study. Latin American, European, and North American maize production systems, which also face DM pressure, were entirely absent from the included literature. This geographic concentration limits the generalisability of the synthesised findings to agroecological contexts not represented in the evidence base and raises questions about whether screening methods validated in South and Southeast Asian environments will perform equivalently elsewhere.

Closely related to the geographic limitation is the imbalance in pathogen species coverage. Although the review’s search strategy and eligibility criteria encompassed all DM pathogen species, the included evidence is dominated by studies evaluating *P. sorghi* and *P. heteropogoni*. Screening methods for other pathogens of economic significance—notably *Sclerophthora rayssiae* var. *zeae* (brown stripe downy mildew), which is prevalent in the Indian subcontinent and relies on soil-borne oospores rather than airborne conidia as the primary infection mechanism—were not specifically validated in any included study. George et al. [6] remains the only study in this review that simultaneously evaluated resistance across five pathogen species, and its finding of a stable chromosome 6 QTL is particularly valuable precisely because the remaining literature is species-restricted. The development and validation of species-specific screening protocols, particularly for *S. rayssiae* and *P. maydis*, constitute an urgent research priority.

Furthermore, the heterogeneity in resistance classification systems across studies complicates cross-study comparison. Although most authors adopted percentage incidence as the primary outcome measure, the thresholds defining resistance categories varied: some studies classified genotypes with less than 10% incidence as resistant, while others used 15% as the cut-off [7,35]. Likewise, ordinal severity scales (e.g., 0–9) were employed by Djaenuddin et al. [36] alongside percentage-based metrics, requiring harmonisation during data extraction. Such methodological inconsistency impedes direct quantitative comparison and may obscure genuine differences in method performance. Likewise, the molecular evidence, while promising, remains limited in scope: only five studies reported QTL or candidate gene data, and the total number of mapping populations evaluated was small. The wide variation in QTL effect sizes across studies (PVE = 12–31% for the chromosome 6 locus) suggests that the genetic architecture of resistance may be population-dependent, necessitating validation in diverse genetic backgrounds before markers can be confidently deployed in breeding programmes [1].

### 4.3. Limitations of the Review Process

The review process itself was subject to several methodological constraints. The restriction to English-language publications may have excluded relevant studies published in Bahasa Indonesian, Hindi, or other regional languages, given that a substantial proportion of DM research originates from non-Anglophone countries. Although the inclusion of CAB Abstracts in the search strategy was intended to capture agricultural literature that might not be indexed in biomedical databases, the extent of language-related omission cannot be quantified. The decision not to conduct a formal meta-analysis, while justified by the pronounced heterogeneity in pathogen species, screening protocols, and outcome reporting conventions, means that the synthesis relies on qualitative pattern recognition rather than pooled effect estimates with statistical precision. This limits the strength of the comparative conclusions that can be drawn regarding the relative superiority of one screening method over another.

Additionally, the small number of included studies (n = 12) constrained the capacity to detect and control for potential confounders through subgroup analysis or meta-regression. The qualitative sensitivity analysis, while informative, cannot fully substitute for the formal heterogeneity diagnostics (e.g., I^2^ statistics, prediction intervals) that would have been possible with a larger and more homogeneous evidence base.

### 4.4. Implications for Practice, Policy, and Future Research

The findings of this review carry several practical implications for maize breeding programmes targeting DM resistance. For resource-constrained programmes in tropical regions, field-based spreader row screening remains the most accessible and widely validated approach, provided that adequate inoculum pressure can be maintained—a condition that should be verified through the inclusion of susceptible check genotypes such as CM500 in every trial [2,7]. Where glasshouse facilities are available, the sandwich inoculation technique offers a valuable complement to field screening, particularly for off-season evaluation or preliminary germplasm characterisation, given its demonstrated concordance with field outcomes [3,7]. Programmes with access to molecular laboratory infrastructure should consider integrating SSR markers flanking the chromosome 3 and chromosome 6 QTLs (e.g., bnlg420, phi073) into early-generation selection schemes, thereby reducing the volume of material that requires resource-intensive field phenotyping [6,33].

From a policy perspective, the heterogeneity in resistance classification systems documented in this review underscores the need for international harmonisation of DM screening protocols. Standardised incidence thresholds, assessment time points, and severity rating scales—developed through collaborative agreement among major breeding centres such as CIMMYT, ICAR, and national agricultural research systems—would substantially improve the comparability of screening outcomes across studies and facilitate the exchange of resistant germplasm between programmes. Investment in multi-environment, multi-pathogen screening networks, analogous to the international nursery systems operated for wheat rusts, would further strengthen the evidence base by enabling the simultaneous evaluation of genotypes across the full spectrum of DM pathogen diversity.

Several priorities emerge for future research. First, there is a pressing need for screening studies conducted in sub-Saharan Africa, Latin America, and other underrepresented regions to determine whether the methods validated in Asian environments generalise to different agroecological and pathogen contexts. In addition, future phenotypic screening studies should adopt blinded outcome assessment protocols in which disease scorers are masked to genotype identity. Where blinding is impractical in field settings, we recommend the following: (i) double-blind scoring, in which two independent assessors unaware of genotype identity evaluate each plot; (ii) digital image-based phenotyping using unmanned aerial vehicles (UAVs) or handheld imaging systems coupled with machine learning-based disease quantification algorithms, which eliminates subjective visual assessment entirely; and (iii) randomised plot labelling systems that conceal genotype identity during scoring. These measures would substantially strengthen the evidence base for cross-method reliability. Similarly, the molecular findings of this review, while encouraging, require independent validation in broader genetic backgrounds and against a wider panel of pathogen isolates before they can be translated into operational marker-assisted selection platforms.

## 5. Conclusions

This systematic review synthesised evidence from 12 peer-reviewed studies to address three research questions on the screening of downy mildew resistance in maize.

Regarding the first question, what are the most commonly used methods to screen maize for downy mildew resistance? The review identified field-based spreader row systems and artificial conidial spray inoculation as the most frequently employed phenotypic screening techniques, reported in seven and six studies, respectively. Controlled-environment methods, particularly the glasshouse sandwich inoculation technique, were used less widely but generated the most uniform and intense disease pressure. Five of the twelve studies supplemented phenotypic evaluation with molecular approaches, primarily QTL mapping using SSR and RFLP markers and gene expression profiling via qRT-PCR.

In response to the second question, how effective and reliable are these screening techniques under different environmental and pathogen conditions? The evidence indicates that both spreader row and sandwich inoculation protocols are highly effective at discriminating resistant from susceptible germplasm, as confirmed by susceptible check incidence of 92–100% across studies. Cross-method correlations were consistently strong (r = 0.92–0.99), demonstrating that resistance rankings are transferable between field and glasshouse environments. Broad-sense heritability estimates ranged from 0.50 to 0.97, indicating that phenotypic variation in DM incidence is substantially genetically determined. Reproducibility, however, was pathogen-dependent: screening against sorghum downy mildew proved more repeatable (88%) than against Rajasthan downy mildew (76%), and CVs varied considerably across multi-site field trials (14–98%). The certainty of evidence for method effectiveness and reliability was rated as moderate under the GRADE framework.

Concerning the third question, what phenotypic or molecular markers are associated with resistance? The percentage disease incidence assessed 21–34 days after inoculation was the universal phenotypic marker, with resistance thresholds typically set at less than 10% incidence. Supplementary phenotypic indicators included suppression of systemic infection, reduced lesion size, and SPAD chlorophyll values. At the molecular level, QTLs on chromosomes 2, 3, and 6 were consistently identified, with the chromosome 6 locus exhibiting the broadest stability across pathogen species and environments. Flanking markers bnlg420 (chromosome 3) and SSR/RFLP markers on chromosome 6 represent the most promising candidates for marker-assisted selection, and candidate genes bZIP33, Bak1, and Ppr warrant further functional validation. The certainty of evidence for marker–resistance associations was rated as low, reflecting primarily study design limitations rather than demonstrated marker instability across populations. We therefore conclude that the low certainty rating is attributable to methodological limitations in the existing evidence base—particularly small sample sizes, absence of analytical pre-registration, and lack of independent validation cohorts—rather than to demonstrated failure of the markers to replicate across populations. Definitive resolution of cross-population marker stability will require validation in broader and more diverse genetic backgrounds.

From a theoretical standpoint, the consistency of chromosome 6 QTL effects across multiple pathogen species supports the hypothesis that basal immunity pathways, rather than narrow race-specific gene-for-gene interactions, may underpin durable DM resistance in maize. This has implications for resistance gene deployment strategies, suggesting that introgression of broadly effective QTLs may confer more lasting protection than pyramiding pathogen-specific resistance genes. Practically, the review supports three specific recommendations: (i) adoption of the glasshouse sandwich technique for off-season preliminary screening; (ii) integration of SSR markers flanking chromosomes 3 and 6 QTLs into early-generation marker-assisted selection; and (iii) mandatory inclusion of CM500 or equivalent susceptible checks in all screening trials to validate inoculation adequacy. From a policy perspective, international harmonisation of DM resistance classification thresholds—developed through collaborative agreement among CIMMYT, ICAR, and national agricultural research systems—would substantially improve the comparability of screening outcomes and facilitate germplasm exchange. Finally, this review identifies an urgent need for additional primary screening studies, particularly in underrepresented regions and against underrepresented pathogen species, to broaden the evidence base upon which breeding decisions depend.

## Figures and Tables

**Table 1:** (**a**): Effect Measures for downy mildew screening in maize. (**b**): Performance metrics for downy mildew screening in maize.

(**a**)			
**Author**	**Outcome**	**Key Effect Measures**	**Significance**
Kling et al. [11]	% DM incidence	Range: 0.1–41.4% (test); 6–94% (checks); LSD = 13.1	*p* < 0.001
Arulselvi & Selvi [3]	% incidence; R < 10%, S > 50%	r = 0.99 (field vs. glasshouse); UMI935(w): 0–2.6%; UMI79: 88–100%	Not reported
Yamada & Aday [34]	% systemic symptoms (28 DAI)	Max R vs. S difference: 50–58%; Optimal: 0.5 leaf + 50 × 10^3^ conidia/mL	L **: F = 77.27, D **: F = 20.31, M **: F = 71.67
Rashid et al. [7]	% incidence; HR = 0%, R = 1–10%, HS = 76–100%	r: SDM-F vs. RDM-F = 0.922 **; h^2^ = 0.65–0.97; CML-433: 0%; 14 lines < 18.8%	G **: *p* < 0.01; G × M *: *p* < 0.05
Yen et al. [9]	% incidence (34 DAI); R < 10%, S > 50%	5 lines R to both (0–8.6%); h^2^: SDM = 0.75, RDM = 0.63; CM500: 99–100%	G **: *p* = 0.0001; Y × G **: *p* = 0.0001
Kim et al. [1]	% incidence; HR = 0%, HS = 76–100%	7 QTLs: qDM1 LOD = 14.12, PVE = 12.95%; qDM4 LOD = 18.16; 15 genes upregulated	LOD ≥ 3.0 (α = 0.05); qRT-PCR: *p* < 0.001
Kim et al. [8]	% incidence; HR-HS scale	Expression: R = 1050–2338%, S = 93–133%; Apr: Ki11 = 0–5%; Sep: Ki11 = 10–25%	*p* = 0.05 (DMRT); r = 0.98, *p* < 0.01
George et al. [6]	% incidence (21–34 DAI)	6 QTLs; Chr6: LR = 56.7 **, R^2^ = 13–31%; h^2^ = 0.50–0.75; Ki3: 0–32%, CML138: 50–100%	G **: *p* = 0.0001; LOD ≥ 3.0
Jadhav et al. [33]	% incidence (30 DAS); SPAD; LW	Chr3 QTL: LOD = 5.3–18.1, PVE = 14–19%; r (FC vs. GC) = 0.949 **; h^2^ = 87–97%	*p* < 0.01 **; LOD ≥ 2.5
Hooda et al. [35]	% incidence; R ≤ 15%, MR = 15–20%, S > 20%	RDM-R: 3 lines (8.6–13.9%); SDM-R: 16 lines; Multi-R: 3 lines	Not reported
Djaenuddin et al. [36]	Severity 0–9; R < 10%, HS > 50%	DM: 20–86%; LB: 0.6–80%; Best: 05022-00847 (20%); 4 acc. < 28%	Qualitative (vs. Nei-9008)
Sumathi et al. [2]	% incidence; R < 10%, S > 50%	16/22 progenies: 0% (R); Range: 0–61.9%; CM500: 100%	Not reported
(**b**)			
**Author**	**Performance Metrics**		
Kling et al. [11]	Check: 6–94%; CV = 31–35%; Incubator <5% escape		
Arulselvi & Selvi [3]	CM500: 92–100%; r = 0.99 method correlation		
Yamada & Aday [34]	Optimal conditions identified; No secondary infection		
Rashid et al. [7]	Check: 93.6–97.2%; 100% HR spill-over; CV = 14–20%		
Yen et al. [9]	SDM: 88% reproducible; RDM: 76%; CV = 19–30%		
Kim et al. [1]	B73/CML270: 100%; All seven QTLs validated; 65.8% genome		
Kim et al. [8]	Checks: 100% (both seasons); five candidates identified		
George et al. [6]	Chr6 stable across all five species; Checks: 85–100%; CV = 15–98%		
Jadhav et al. [33]	Chr3 QTL: 3/3 env (100%); bnlg420 marker validated		
Hooda et al. [35]	CM500: 100%; three lines consistent 2 years; Strain variation		
Djaenuddin et al. [36]	Spreader: >80% infection; 2 weeks to symptom		
Sumathi et al. [2]	CM500: 100%; 3:30–4:30 AM inoculation; Oospore soil		

**Key:** *p* = Phenotypic; Both = Phenotypic + Molecular; R = Resistant; S = Susceptible; HR = Highly Resistant; HS = Highly Susceptible; MR = Moderately Resistant; DM = Downy Mildew; DAI = Days After Inoculation; DAS = Days After Sowing; acc. = accessions; env = environments; L = Leaf stage; D = Density; M = Material; G = Genotype; ** = *p* < 0.01; * = *p* < 0.05.

**Table 2:** Summary of excluded studies at the full-text assessment stage.

Reason for Exclusion	Operational Definition	Examples of Excluded Studies
Wrong population	Studies focusing on non-maize crops, mixed crops where maize resistance screening was not the primary focus, or model organisms.	Anahosur & Hegde [19]; Carlsson et al. [21]; Madhu et al. [37]; Narayana et al. [22]; Odvody & Frederiksen [16]; Mushayi et al. [13]; Susilowati et al. [15]
Wrong study design	Reviews, methodological papers, guidelines, tools, or conceptual papers without primary field-based phenotypic data.	Frederiksen & Renfro [38]; Haddaway et al. [24]; Higgins et al. [30]; Jenkins [26]; Li et al. [27]; Ouzzani et al. [28]; Page et al. [24]; Rethlefsen et al. [25]; Sterne et al. [32]; Waffenschmidt et al. [29].
Wrong outcome	Studies addressing disease epidemiology, genetic mapping, QTL analysis, or molecular genetics, rather than a systematic evaluation of screening methods.	Agrama et al. [23]; Bock et al. [20]; Carlsson et al. [21]; George et al. [6]; Kim et al. [8]; Kim et al. [1]; Rashid et al. [18]; Yen et al. [9].

**Table 3:** Characteristics of included studies on screening methods for downy mildew resistance in maize.

Author Name	Region	Screening Methods	Effectiveness Under Different Environmental Conditions	Reliability Under Different Pathogen Conditions	Phenotypic Markers Associated with Resistance	Molecular Markers Associated with Resistance
Kling, et al. [11]	Nigeria	Field and spray inoculation; seedling/incubator methods; spreader rows	Conidia need >85% RH and ~20–21 °C; resistance holds across environments	Resistance observed across strains, but can shift with strain changes	Systemic infection suppressed; lower grain loss; better vigour	Polygenic control; gene interactions
Arulselvi and Selvi [3]	India	Field sick-plot; glasshouse	Field works with natural inoculum; the glasshouse most efficient overall	Depends on natural field inoculum levels	UMI 935(w) resistant	Nil
Rashid, et al. [7]	India	Sandwich (glasshouse); infector row and whorl (field)	Sandwich yields the highest, consistent pressure; field varies by site	Consistent vs. *P. sorghi & P. heteropogoni*; field virulence varies	Disease score; % infection (<10% for resistant)	QTLs include loci common to SDM/RDM.
Yen, et al. [9]	India	Sandwich (SDM); whorl inoculation (RDM) under field	Effective in tropical/subtropical sites (Mandya, Udaipur)	SDM-resistant lines often RDM-resistant; RDM-resistant show variable SDM	% incidence classes (R, MR, MS, S)	QTLs overlapping for SDM/RDM.
Kim, et al. [1]	Cambodia	qRT-PCR; QTL analysis	Incidence tracked across wet/cool vs. dry months	QTLs mapped for *P. sorghi*, *P. maydis*, *S. macrospora*	Upregulated genes in resistant genotypes	Markers incl. umc1165, bnlg1297, umc2353, phi098
Kim, et al. [8]	Cambodia	Spreader rows; QTL; RT-PCR	High resistance in CML228, Ki3, Ki11 under suitable conditions	Reliable when the environment favours disease	Low symptoms in resistant germplasm	bZIP33, Bak1, Ppr candidates
George, et al. [6]	India	Spreader rows; artificial and whorl inoculation; modified spreader rows	Tested at five Asian sites with varied climates	QTL effects varied by strain/site; chr-6 QTL broad	% disease incidence; resistant check Ki3; susceptible CML138	SSR/RFLP; QTL on chr-6 tightly linked
Jadhav, et al. [33]	India	Spreader rows; seedling spray; artificial epiphytotic	Stable across field and glasshouse (Coimbatore, Mandya)	QTLs stable though pathogen effect varied	PDI, SPAD, leaf width	SSRs; bnlg420 (chr-3), phi073
Djaenuddin et, al., [36]	Indonesia	Field under natural pressure; artificial inoculation; severity scoring; marker analysis	Multi-region Indonesia trials include greenhouse and field trials	Consistent patterns; some accessions highly resistant across environments	% incidence; lesion size; chlorosis; severity	SSRs/SNPs; loci on chr-2 and chr-6
Sumathi, Ganesan & Senthil [2]	India	Spreader rows; artificial conidial inoculation; severity scoring	Rabi 2013 sick-plot maintained high pressure	100% infection in susceptible checks confirmed pressure	% incidence; R/MR/S classes	Nil
Yamada, M. & Aday, A. [34]	Philippines	Artificial inoculation + field screening	Designed for humid tropical epiphytes	High consistency with local isolates	Delayed symptoms; smaller lesions; limited sporulation	Nil
Hooda, et al. [35]	India	Field at 3 hotspots; artificial spore sprays; standardised scoring	Effective across humid to semi-arid sites; some lines broadly resistant	Consistent reactions vs. *P. sorghi & P. heteropogoni*	Low severity; minimal systemic infection/chlorosis	Nil

**Table 4:** Risk of bias assessment.

Author and Year	D1: Bias Arising from the Randomisation Process	D2: Bias Due to Deviation from Intended Interventions	D3: Bias Due to Missing Outcome Data	D4: Bias in Measurement of the Outcome	D5: Bias in Selection of the Reported Result	Overall Risk of Bias
Kling et al. [11]	Some concerns	Low	Low	Some concerns	Low	Some concerns
Arulselvi & Selvi, [3]	Low	Low	Low	Some concerns	Low	Low
Yamada, M., & Aday, A. [34]	Some concerns	Low	Low	Some concerns	Low	Some concerns
Rashid et al. [7]	Some concerns	Low	Low	Some concerns	Some concerns	Some concerns
Yen et al. [9]	Low	Low	Some concerns	Some concerns	Some concerns	Some concerns
Kim et al. [1]	Some concerns	Low	Some concerns	Some concerns	Some concerns	Some concerns
Kim et al. [8]	Some concerns	Low	Some concerns	Some concerns	Some concerns	Some concerns
George et al. [6]	Low	Low	Low	Some concerns	Some concerns	Some concerns
Jadhav et al. [33]	Low	Low	Low	Some concerns	Some concerns	Some concerns
Hooda et al. [35]	Some concerns	Low	Low	Some concerns	Some concerns	Some concerns
Djaenuddin et al. [36]	Some concerns	Low	Low	Some concerns	Some concerns	Some concerns
Sumathi, Ganesan & Senthil [2]	Some concerns	Low	Low	Some concerns	Some concerns	Some concerns

**Table 5:** Physical map positions of QTLs associated with downy mildew resistance in maize, aligned to the b73 refgen_v5 assembly.

QTL/Locus	Chr	Bin	Flanking Markers	Physical Position (Mb) ^1^	LOD	PVE (%)	Pathogen Species Tested	Genomic Interval Overlap Assessment	Study
DM-QTL6	6	6.01	bnlg1361—umc1006	~4.5–25.0	≥3.0 ^2^	13–31	*P. sorghi*, *P. heteropogoni*, *P. maydis*, *P. philippinensis*, *S. macrospora*	Core interval; overlaps with Djaenuddin et al. [36] chr-6 locus inbin 6.01–6.02	George et al. [6]
DM-QTL (minor loci)	1, 2, 3, 7, 10	Various	Various SSR/RFLP	Not precisely resolved	<3.0	<10	*Variable across species*	Minor; not consistently detected across studies	George et al. [6]
qSDM3	3	3.05–3.06	bnlg420—phi073	~155.3–165.8	5.3–18.1	14–19	*P. sorghi*	Distinct region; no overlap with chr-6 loci. Independently validated across 3 environments (Coimbatore, Mandya, field + glasshouse)	Jadhav et al. [33]
qDM1	1	1.02–1.03	phi098—umc1165	~4.6–18.5	14.12	12.95	*P. sorghi*, *P. maydis*, *S. macrospora*	No overlap with chr-6 or chr-3 QTLs; novel region on chr-1	Kim et al. [1]
qDM4	4	4.05–4.06	bnlg1297—umc2353 ^3^	~148.2–168.0	18.16	NR	*P. sorghi*, *P. maydis*, *S. macrospora*	Highest LOD in study; no overlap with the chr-6 region	Kim et al. [1]
qDM2–qDM7	2, 3, 5, 6, 9	Various	Multiple SSR loci	Variable ^4^	3.0–12.5	5.2–11.8	*P. sorghi*, *P. maydis*, *S. macrospora*	qDM6 (chr-6) may overlap with George et al. bin 6.01 interval; requires fine-mapping to confirm	Kim et al. [1]
DM-chr6	6	6.01–6.02	SSR/SNP panel	~4.5–90.0 ^5^	NR	NR	*P. philippinensis*	Substantial positional overlap with George et al. [6] chr-6 QTL in bin 6.01; likely same underlying locus	Djaenuddin et al. [36]
DM-chr2	2	2.04–2.05	SSR/SNP panel	~15.0–45.0 ^5^	NR	NR	*P. philippinensis*	Partially overlaps minor chr-2 signal in George et al. [6]; independent confirmation needed	Djaenuddin et al. [36]
SDM/RDM shared loci	6 (putative)	NR	Not specified	Not mapped to physical coordinates	NR	NR	*P. sorghi*, *P. heteropogoni*	Positional data insufficient for overlap assessment; SDM/RDM co-resistance reported phenotypically	Rashid et al. [7]
SDM/RDM overlapping QTLs	NR	NR	Not specified	Not mapped to physical coordinates	NR	NR	*P. sorghi*, *P. heteropogoni*	Phenotypic co-segregation reported; no marker-based physical mapping available	Yen et al. [9]

^1^ Physical positions are approximate, cross-referenced from MaizeGDB (https://www.maizegdb.org) using the B73 RefGen_v5 assembly. Where original studies reported only genetic (cM) positions or bin locations, physical coordinates were estimated from the nearest mapped flanking markers. Positions should be considered indicative rather than definitive. ^2^ George et al. [6] reported a likelihood ratio of 56.7 (*p* < 0.01) rather than a conventional LOD score; the LOD threshold of ≥3.0 was applied for QTL declaration. ^3^ Marker assignments to chromosomes follow the original study; bnlg1297 and umc2353 positions reflect the RIL mapping population used by Kim et al. [1] and may differ from physical positions in other genetic backgrounds. ^4^ Individual physical positions for qDM2–qDM7 are available in the original publication (Kim et al., [1]) ^5^ Djaenuddin et al. [36] used a genome-wide SSR/SNP panel; the broad interval reflects the resolution of the marker set rather than a single large-effect QTL. NR = Not reported in the original study.

## Data Availability

No new data were created or analysed in this study. Data sharing is not applicable to this article.

## Supplementary Materials

The following supporting information can be downloaded at: https://www.mdpi.com/article/10.3390/genes17030350/s1.

## Abbreviations

The following abbreviations are used in this manuscript:

DM	Downy Mildew
MAS	Marker-Assisted Selection
MeSH	Medical Subject Headings
MR	Moderately Resistant
OPVs	Open-Pollinated Varieties
PRISMA	Preferred Reporting Items for Systematic Reviews and Meta-Analyses extension for searching
QTL	Quantitative Trait Loci
R	Resistant
RDM	Rajasthan Downy Mildew
RFLPs	Fragment Length Polymorphisms
ROBINS-I	Risk Of Bias In Non-randomised Studies—of Interventions
SDM	Sorghum Downy Mildew
SNP	Single-nucleotide polymorphism
SSRs	Simple Sequence Repeats
