# Supplementary material for: Screening Methods for Downy Mildew Resistance in Maize: A Systematic Review
Source: Genes (Basel). 2026 Mar 22;17(3):350. doi: 10.3390/genes17030350 (PMC13026945; doi:10.3390/genes17030350)
Supplement: Supplementary file 1 [file genes-17-00350-s001.zip › prisma flow.pdf]

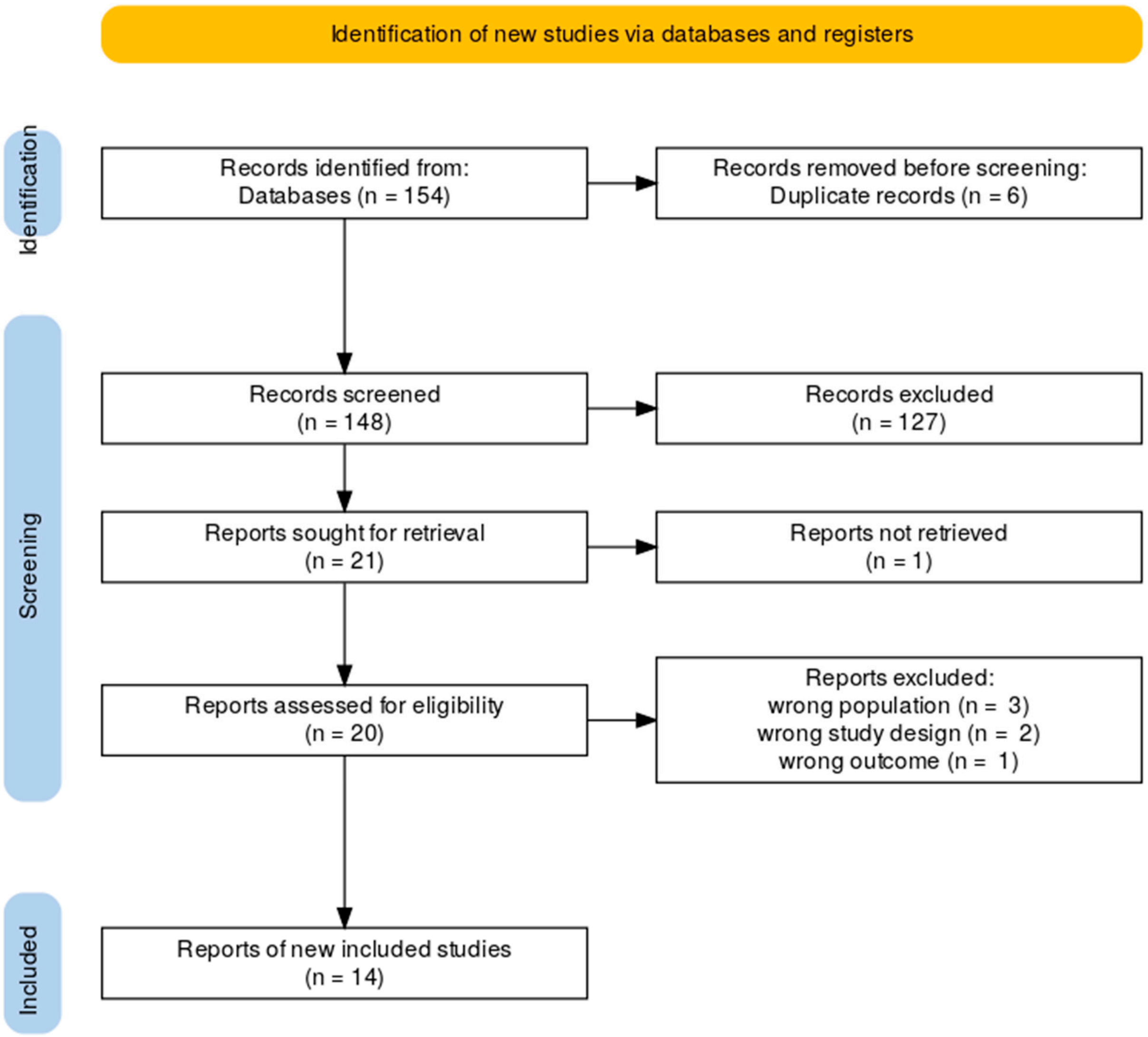
Identification of new studies via databases and registers
Identification
Records identified from:
Databases (n = 154)
Records removed before screening:
Duplicate records (n = 6)
Screening
Records screened
(n = 148)
Records excluded
(n = 127)
Reports sought for retrieval
(n = 21)
Reports not retrieved
(n = 1)
Reports assessed for eligibility
(n = 20)
Reports excluded:
wrong population (n = 3)
wrong study design (n = 2)
wrong outcome (n = 1)
Included
Reports of new included studies
(n = 14)
